# Development and validation of the healthcare provider and family bidirectional digital communication scale

**DOI:** 10.1371/journal.pone.0338410

**Published:** 2025-12-04

**Authors:** Yu-Chun Diao, Meei-Fang Lou

**Affiliations:** 1 School of Nursing, College of Medicine, National Taiwan University, Taipei, Taiwan; 2 Department of Nursing, National Taiwan University Hospital, Taipei, Taiwan; NED University of Engineering and Technology, PAKISTAN

## Abstract

**Introduction:**

Effective communication between healthcare providers and families is critical in ICUs, yet existing assessment scales predominantly focus on unidirectional, face-to-face interactions. The COVID-19 pandemic accelerated adoption of digital communication modalities that have persisted in contemporary practice. This study developed and validated the Healthcare Provider and Family Bidirectional Digital Communication Scale (HF-BDCS) to address these gaps.

**Materials and Methods:**

The HF-BDCS was developed through literature review and qualitative interviews with 15 stakeholders (healthcare providers and family members). Content validity was assessed by 5 experts. Psychometric evaluation with 300 participants (100 physicians, 100 nurses, 100 family members) included exploratory factor analysis and internal consistency assessment using Cronbach’s alpha.

**Results:**

Expert review yielded 14 items with excellent content validity (S-CVI/Ave: relevance=1.00, importance=1.00, clarity=0.98). Exploratory factor analysis produced a 13-item scale with three factors explaining 64.53% of variance: Digital Communication Efficiency and Quality (6 items, 25.87%), Digital Communication Perceptions (5 items, 25.12%), and Digital Communication Regulations (2 items, 13.54%). Internal consistency was good (Cronbach’s alpha=0.80-0.86) across all factors and participant subgroups.

**Discussion:**

The HF-BDCS is the first validated bidirectional instrument for assessing digital communication in ICUs, demonstrating strong psychometric properties. By capturing both healthcare provider and family perspectives, the scale enables identification of perception discrepancies and supports improvements in digital communication practices, ultimately enhancing patient- and family-centered care in contemporary ICUs.

## Introduction

The COVID-19 pandemic fundamentally transformed intensive care units (ICUs) communication practices. Stringent visitation restrictions necessitated a shift from face-to-face to digital communication, compelling healthcare providers to adopt video conferencing, messaging applications, and telephone calls to maintain connections with families [1,2]. The pandemic accelerated adoption of diverse digital platforms. This transformation has persisted post-pandemic, with digital communication becoming integral to contemporary ICU practice [3].

Effective communication between healthcare providers and families is fundamental to ICU care quality, influencing family psychological well-being and patient outcomes [1]. Digital communication offers important advantages: overcoming temporal and spatial constraints, enabling participation of dispersed family members, and supporting bidirectional exchanges where both parties share information and participate actively in care discussions [3].

Effective communication is essential for ICU care quality and family satisfaction [4]. Several validated scales exist for evaluating ICU communication, including family-focused instruments such as the Critical Care Family Needs Inventory (CCFNI, 45 items) [5,6]. Society of Critical Care Medicine Family Needs Assessment (SCCMFNA, 14 items) [7]. Critical Care Family Satisfaction Survey (CCFSS, 20 items) [8], and Family Satisfaction in the Intensive Care Unit scale (FS-ICU, 34 items) [9]. These tools serve as unidirectional mechanisms for healthcare teams to assess family needs and satisfaction. Tools assessing healthcare provider communication also exist but lack extensive validation and similarly capture only single perspectives.

However, existing scales have significant limitations. First, their unidirectional nature assesses communication from only one perspective. In clinical practice, healthcare providers and families often hold differing perceptions of the same communication exchange, yet unidirectional assessment cannot capture these discrepancies. Effective ICU communication requires bidirectional exchanges where both parties express perspectives and identify Perceptions gaps to achieve mutual understanding. Second, existing instruments were designed for face-to-face interactions and cannot adequately evaluate digital communication modalities, which overcome temporal and spatial constraints to enable participation regardless of location or time restrictions [1]. Consequently, no validated instrument exists to assess bidirectional digital communication effectiveness from both healthcare provider and family perspectives.

In this study, digital communication refers to technology-mediated exchanges—including telephone calls, messaging applications, and video conferencing—used to convey clinical information and emotional support between healthcare providers and families. Bidirectional digital communication specifically denotes interactive exchanges enabling both parties to share information, clarify understanding, and provide timely responses, with communication effectiveness evaluated from both perspectives simultaneously. To address this gap, we developed and validated the Healthcare Provider and Family Bidirectional Digital Communication Scale (HF-BDCS).

### Aims and objectives

The aim of this study was to develop and validate the HF-BDCS for assessing digital communication effectiveness between healthcare providers and families in ICUs.

The specific objectives were to:

1. Develop scale items to capture bidirectional digital communication effectiveness between healthcare providers and families in ICUs.
2. Establish content validity of the developed scale.
3. Evaluate psychometric properties including construct validity through exploratory factor analysis and internal consistency reliability assessment.
4. Examine the scale performance across different participant groups (healthcare providers and families).

### Methods

This study followed a systematic process for developing and validating the Healthcare Provider and Family Bidirectional Digital Communication Scale (HF-BDCS). Scale development procedures followed best practices for instrument development as outlined by DeVellis and Thorpe [10]. The development process comprised two main phases: (1) item development and (2) psychometric evaluation.

### Design

This study adopted a two-phase sequential exploratory mixed-methods design [11,12], combining qualitative inquiry (Phase 1) to inform quantitative instrument development and validation (Phase 2) [10].

Unlike the Delphi technique, which primarily relies on expert consensus [13], this approach incorporated firsthand experiences from 15 participants (5 physicians, 5 nurses, and 5 family members) to ensure authentic user perspectives. This design has established precedent in developing family-reported outcomes in critical care [14].

#### Phase 1: Item Development.

An initial pool of items was derived from a comprehensive literature review and semi-structured interviews exploring digital communication between healthcare providers and families in ICUs. The preliminary items aimed to capture a broad range of communication attributes without predefined domains, allowing the factor structure to emerge empirically from the data. Content validity was assessed by a five-member expert panel (1 physician, 1 nurse, 2 researchers, 1 family member with ICU digital communication experience).

#### Phase 2: Psychometric Evaluation.

Construct validity was examined through exploratory factor analysis (EFA) using principal component analysis with Varimax rotation and Kaiser normalization, and reliability was assessed using Cronbach’s alpha. Large-sample data collection (n = 300; 100 physicians, 100 nurses, 100 family members) ensured robust psychometric testing. This two-phase process ensured both theoretical grounding and empirical rigor in developing the HF-BDCS.

### Scale development process

#### Item generation.

The item generation process incorporated multiple evidence sources following best practices for scale development [10].

Literature Review: Relevant literature was identified through PubMed, CINAHL, and Google Scholar using search terms: “intensive care,” “healthcare provider,” “family,” and “digital communication.” Fifteen papers published between 2015–2023 addressing healthcare provider family communication in ICU settings were reviewed to identify relevant communication aspects such as information exchange, timeliness, emotional support, and technology usability, which informed interview guide development.

Qualitative Interviews: Informed by literature themes and the primary investigator’s 15 years of ICU nursing experience implementing digital communication, a semi-structured interview guide was developed to explore perspectives, experiences, and satisfaction with digital communication. As described in Design, 15 participants were purposively selected. Interviews were transcribed and analyzed to identify salient communication themes.

Item Generation: Based on synthesis of interview findings, literature themes, and clinical observations, 17 preliminary items were generated to capture key aspects of digital communication effectiveness without predefined factor structure, allowing domains to emerge empirically. These multiple sources ensured items reflected both theoretical insights from literature and authentic stakeholder experiences from interviews and clinical practice. Items were rated on a 5-point Likert scale (1 = strongly disagree to 5 = strongly agree). The 17-item pool underwent expert content validity assessment.

In this study, digital communication was defined as bidirectional interaction between healthcare providers and patients’ families through technology-mediated channels (telephone, messaging applications, video conferencing) rather than face-to-face communication, supporting timely, reciprocal information exchange in ICUs. This multi-source approach ensured both theoretical grounding from existing literature and practical relevance from real-world digital communication experiences in critical care contexts.

### Psychometric evaluation

#### Content validity.

Content validity was assessed following established guidelines for scale development [15,16] through a two-round evaluation process. As described in the Design section, five experts with diverse expertise evaluated all preliminary items. Round 1: Each expert independently rated all 17 items on three dimensions (relevance, importance, and clarity) using a 4-point scale (1 = low, 2 = moderate, 3 = high, 4 = very high) [15]. Round2: Items were revised based on Round 1 feedback and re-evaluated by the same experts for dimensions that did not meet validity standards.

Content Validity Index (CVI) was calculated following established methods [16,17]. Item-level CVI (I-CVI) was computed as the proportion of experts rating 3 or 4 (indicating high relevance/ importance/clarity) on each dimension. Scale-level CVI (S-CVI/Ave) was calculated as the average of all I-CVIs across items. Following recommended standards [15], items achieving I-CVI ≥ 0.80 on all three dimensions were retained. Items with I-CVI < 0.80 were revised based on expert qualitative feedback or deleted if deemed not essential.

#### Construct validity.

Construct validity was assessed through exploratory factor analysis (EFA) following established procedures [18,19]. Data suitability was verified using Kaiser-Meyer-Olkin (KMO) measure and Bartlett’s test of sphericity [20]. Principal component analysis with orthogonal Varimax rotation and Kaiser normalization was performed to achieve interpretable factor structure [21, 22]. Varimax rotation maximizes variance of squared loadings, creating clearer factor distinction [22].

The number of factors was determined using multiple criteria [23]: (1) eigenvalues >1.0, (2) scree plot inspection, and (3) conceptual interpretability. Items with factor loadings ≥0.50 were retained; items with cross-loadings ≥0.30 were examined for removal [21]. To examine scale applicability across stakeholder groups, separate exploratory factor analyses were conducted for healthcare providers (n = 200) and family members (n = 100).

#### Reliability analysis.

Internal consistency reliability was assessed using Cronbach’s alpha [24] for each subscale. Item-subscale correlations were calculated using Pearson’s correlation to assess item discrimination within each subscale. Following established standards [25], alpha values ≥0.70 were considered acceptable. Separate reliability analyses were conducted for healthcare providers and family members to examine consistency across participant groups.

#### Participants.

As described in the Design section, 300 participants were recruited for Phase 2 psychometric testing: 100 physicians, 100 nurses, and 100 family members from medical and surgical ICUs at a tertiary medical center. Participants were selected through purposive sampling based on their experience with digital communication during ICU care. All participants provided informed consent before participation. This sample is distinct from the 15 participants in Phase 1 qualitative interviews and the 5-member expert panel.

### Ethical statement

Ethical approval was obtained from the Research Ethics Committee C of National Taiwan University Hospital (IRB No. 202207043RINC). All participants provided written informed consent before participation.

### Data collection

Data for Phase 2 psychometric evaluation were collected from August 2023 to May 2024. The primary investigator visited each ICU to recruit participants based on inclusion criteria. Eligible physicians, nurses, and family members were individually invited to complete the paper-based questionnaire on-site. The questionnaire took approximately 5–10 minutes to complete. All participants received a gift voucher (approximately USD 3) as appreciation for their time.

## Results

The psychometric analyses demonstrated strong evidence for the validity and reliability of the HF-BDCS. The results are presented according to the evaluation of content validity, construct validity, and internal consistency reliability.

### Content validity

Five experts evaluated 17 preliminary items through a two-round process.

Round 1: All items achieved I-CVI ≥ 0.80 for relevance (S-CVI/Ave = 1.00) and importance (S-CVI/Ave = 1.00). However, clarity was below threshold (S-CVI/Ave = 0.68). Based on expert feedback, items 13–15 were merged into one item due to redundancy, items 16–17 were consolidated due to similarity, and remaining item wording was revised for clarity. This resulted in 14 items.

Round 2: The revised 14 items were re-evaluated for clarity. All items achieved I-CVI ≥ 0.80, with S-CVI/Ave improving to 0.98.

The final 14-item scale demonstrated excellent content validity across all dimensions (relevance: 1.00, importance: 1.00, clarity: 0.98).

### Psychometric evaluation

#### Participant characteristics.

The psychometric evaluation included 300 participants: 100 physicians, 100 nurses, and 100 family members (response rates: 100% for healthcare providers, 91.7% for family members).

Physicians (79% male, mean age 29.6 years) and nurses (91% female, mean age 37.5 years) were predominantly bachelor’s degree holders (96% and 89%, respectively). Family members (55% male, mean age 50.7 years, primarily adult children) had diverse educational levels ranging from high school to graduate degrees. All participants had experience using digital communication during ICU care.

#### Construct validity.

Exploratory factor analysis was conducted on 14 items with 300 participants. Initial data suitability was confirmed (KMO = 0.877, Bartlett’s χ² = 1834.83, p < 0.001). Principal component analysis with Varimax rotation identified a 3-factor solution based on eigenvalues >1.0, scree plot inspection, and conceptual interpretability. One item was removed due to low factor loading (<0.50), resulting in 13 items. Items were renumbered sequentially within their respective factors (Table 1). The final 13-item solution demonstrated excellent data adequacy (KMO = 0.883, Bartlett’s χ² = 1810.79, p < 0.001). All retained items had factor loadings ≥0.50 with no substantial cross-loadings. The three factors explained 64.53% of total variance: – Factor 1 “Digital Communication Efficiency and Quality” (items 1–6): 25.87% of variance – Factor 2 “Digital Communication Perceptions” (items 7–11): 25.12% of variance – Factor 3 “Digital Communication Regulations” (items 12–13): 13.54% of variance. Subgroup exploratory factor analyses for healthcare providers (n = 200) and family members (n = 100) yielded similar 3-factor structures, explaining 63.04% and 68.16% of variance respectively, confirming the scale’s applicability across stakeholder groups.

**Table 1:** Factor Structure of the HF-BDCS (N = 300).

Factor/ Item	Factor Loading	Eigenvalue	Explained Variance
**Factor 1: Digital communication efficiency and quality**		3.36	25.87%
** 1. Digital communication methods eliminate the need for in-person hospital visits, making communication more convenient for both parties.**	0.76		
** 2. Digital communication methods allow both parties sufficient time for medical communication outside of fixed visiting hours.**	0.80		
** 3. Because digital communication methods do not require face-to-face interaction, they reduce stress for both parties.**	0.79		
** 4. Digital communication methods facilitate communication between multiple family members and the healthcare providers.**	0.52		
** 5. When changes in a patient’s condition require unexpected medical decisions, digital communication methods facilitate faster medical decision-making.**	0.66		
** 6. Digital communication methods can be as effective as face-to-face communication.**	0.63		
**Factor 2: Digital communication perceptions**		3.27	25.12%
** 7. Through digital communication methods, both parties can adequately understand and provide information regarding the patient’s medical treatment during hospitalization.**	0.59		
** 8. Digital communication methods enable both parties to express and understand the content of a message.**	0.74		
** 9. Through digital communication methods, both parties can listen patiently to each other.**	0.82		
**10. Through digital communication methods, both parties can actively express care or feel cared for.**	0.77		
**11 Through digital communication methods, both parties can communicate with a positive attitude.**	0.68		
**Factor 3: Digital communication regulations**		1.76	13.54%
**12. The hospital provides clear information on regulations regarding digital communication between healthcare providers and families.**	0.87		
**13. I know how to use the hospital’s digital communication tools (e.g., LINE app, phone calls, online video calls, or other communication software).**	0.89		

Total explained variance = 64.53%.

HF-BDCS: Healthcare Provider and Family Bidirectional Digital Communication Scale.

#### Reliability analysis.

The HF-BDCS demonstrated good to excellent internal consistency (Table 2). Cronbach’s alpha coefficients for all participants were 0.86 for Factor 1 (Digital Communication Efficiency and Quality), 0.84 for Factor 2 (Digital Communication Perceptions), and 0.80 for Factor 3 (Digital Communication Regulations), all exceeding the 0.70 threshold for acceptable reliability.

**Table 2:** Internal Consistency of the HF-BDCS.

Factor	Cronbach’s α		
	All	Healthcare Providers	Families
**Factor 1: Digital communication efficiency and quality**	0.86	0.84	0.84
**Factor 2: Digital communication perceptions**	0.84	0.82	0.91
**Factor 3: Digital communication regulations**	0.80	0.84	0.70

HF-BDCS: Healthcare Provider and Family Bidirectional Digital Communication Scale.

Item-subscale correlations ranged from 0.68 to 0.93 (all p < 0.001, Table 3), indicating that items were appropriately associated with their respective subscales and supporting good item discrimination. Subgroup reliability analyses showed that Cronbach’s alpha coefficients ranged from 0.82 to 0.84 for healthcare providers and from 0.70 to 0.91 for family members across the three factors (Table 2), confirming acceptable to excellent internal consistency across participant groups.

**Table 3:** Correlation Between Items and Subscale Total Scores of the HF-BDCS (N = 300).

Item No.	Factors and Items	r value
**Factor 1: Digital communication efficiency and quality**		
**1**	Digital communication methods eliminate the need for in-person hospital visits, making communication more convenient for both parties.	0.75^**^
**2**	Digital communication methods allow both parties sufficient time for medical communication outside of fixed visiting hours.	0.82^**^
**3**	Because digital communication methods do not require face-to-face interaction, they reduce stress for both parties.	0.76^**^
**4**	Digital communication methods facilitate communication between multiple family members and the healthcare providers.	0.69^**^
**5**	When changes in a patient’s condition require unexpected medical decisions, digital communication methods facilitate faster medical decision-making.	0.78^**^
**6**	Digital communication methods can be as effective as face-to-face communication.	0.78^**^
**Factor 2: Digital communication perceptions**		
**7**	Through digital communication methods, both parties can adequately understand and provide information regarding the patient’s medical treatment during hospitalization.	0.71^**^
**8**	Digital communication methods enable both parties to express and understand the content of a message.	0.81^**^
**9**	Through digital communication methods, both parties can listen patiently to each other.	0.87^**^
**10**	Through digital communication methods, both parties can actively express care or feel cared for.	0.83^**^
**11**	Through digital communication methods, both parties can communicate with a positive attitude.	0.68^**^
**Factor 3: Digital Communication Regulations**		
**12**	The hospital provides clear information on regulations regarding digital communication between providers and families.	0.93^**^
**13**	I know how to use the hospital’s digital communication tools (e.g., LINE app, phone calls, online video calls, or other communication software).	0.90^**^

** *p* < 0.001.

HF-BDCS: Healthcare Provider and Family Bidirectional Digital Communication Scale.

## Discussion

This study developed and validated the 13-item Healthcare Provider and Family Bidirectional Digital Communication Scale (HF-BDCS) comprising three factors: Digital Communication Efficiency and Quality, Digital Communication Perceptions, and Digital Communication Regulations. The scale demonstrated excellent content validity (S-CVI/Ave: relevance = 1.00, importance = 1.00, clarity = 0.98) and good reliability (Cronbach’s alpha = 0.80–0.86) across healthcare provider and family subgroups. The three-factor structure explained 64.53% of total variance.

### Psychometric properties of the HF-BDCS

The HF-BDCS demonstrated strong psychometric properties, meeting or exceeding established standards for scale development [15,16,25]. Content validity was strong, confirming appropriate representation of digital communication dimensions identified through literature review, qualitative interviews, and expert validation.

The three-factor structure distinguishes practical advantages (convenience, temporal flexibility, stress reduction, multi-party communication, faster decision-making, and effectiveness), interpersonal communication quality (understanding, expression, listening, care, and positive attitudes), and institutional support (regulatory clarity and usage knowledge). This structure indicates that effective digital communication depends on technological benefits, interpersonal quality, and institutional support.

Factor structure stability across healthcare providers and families supports bidirectional applicability. Internal consistency was good [25] across all factors and participant groups, indicating robust measurement properties essential for comparing perspectives across stakeholder groups.

### Contributions of the HF-BDCS: Addressing gaps in ICU communication assessment

The HF-BDCS addresses two critical limitations evident in existing ICU communication assessment tools [5–9].

First, the bidirectional design represents an important methodological advancement. While existing scales provide valuable information about family needs and satisfaction, their unidirectional approach cannot reveal whether healthcare providers and families perceive communication similarly. By enabling simultaneous assessment of both perspectives, the HF-BDCS can identify Perceptions discrepancies essential for targeted communication improvements.

Second, the scale responds to the growing importance of digital communication in ICU practice. The COVID-19 pandemic dramatically expanded the role of digital modalities in ICU communication [1,3,26,27]. However, existing instruments were developed for face-to-face interactions and do not capture dimensions unique to technology-mediated exchanges. The HF-BDCS fills this gap by assessing aspects specific to digital communication—such as convenience, temporal flexibility, and regulatory clarity—that are increasingly relevant to contemporary ICU practice.

### Clinical and practical implications

The HF-BDCS has important implications for improving digital communication practices in contemporary ICU settings, with distinct benefits for healthcare providers and families.

For healthcare providers, the scale provides actionable metrics for quality improvement. While healthcare providers appreciate digital efficiency, they often struggle with increased workload, technical barriers, and conveying emotional support via digital platforms [28]. The scale’s bidirectional metrics help teams identify specific communication challenges and guide providers to address families’ needs for assurance and information [28,29]. These data can inform quality improvement initiatives and training programs for digital communication competencies [30].

From a family perspective, the scale represents an important advance in patient- and family-centered care. Families prioritize information, support, and proximity [28], yet digital communication can lead to feelings of isolation [29]. Rather than positioning families solely as information recipients, the HF-BDCS recognizes them as partners whose evaluations are equally important. This validation empowers families to advocate for their needs [28,30] and encourages healthcare providers to strengthen relationship-centered care [29].

### Study limitations

This study has limitations. First, data collection at a single center may limit generalizability. Second, the cross-sectional design does not assess temporal changes or clinical outcomes. Third, criterion validity was not established. Future research should validate the scale across multiple sites and populations, examine longitudinal properties, and establish relationships with objective communication measures and clinical outcomes.

## Conclusions

This study developed and validated the HF-BDCS, the first bidirectional instrument to assess digital communication effectiveness between healthcare providers and families in ICU settings. The scale addresses critical limitations of existing unidirectional, face-to-face focused instruments and demonstrated strong psychometric properties. The HF-BDCS benefits both stakeholder groups. Healthcare providers can identify perception discrepancies and develop targeted communication improvements. Families gain a structured means to assess their experiences and advocate for their communication needs. The bidirectional design facilitates collaborative problem-solving to enhance mutual understanding and satisfaction. As digital communication becomes integral to ICU practice, the HF-BDCS provides a validated tool for systematically evaluating and improving communication quality from both perspectives, supporting patient- and family-centered care principles.

## Supporting information

S1 Questionnaire Healthcare Provider and Family Bidirectional Digital Communication Scale (HF-BDCS). This supplementary file contains the complete 13-item version of the HF-BDCS, including instructions and scoring information, used for psychometric testing.
(DOCX)
