# Supplementary material for: Development and validation of the healthcare provider and family bidirectional digital communication scale
Source: PLoS One. 2025 Dec 4;20(12):e0338410. doi: 10.1371/journal.pone.0338410 (PMC12677555; doi:10.1371/journal.pone.0338410)
Supplement: S1 Questionnaire — This supplementary file contains the complete 13-item version of the HF-BDCS, including instructions and scoring information, used for psychometric testing. (DOCX) [file pone.0338410.s001.docx]

This questionnaire, titled "Health Provider–Family Bidirectional Digital Communication Scale (HF-BDCS)," was developed by the authors of the manuscript submitted to PLOS ONE.

The authors affirm that they are the original creators of this instrument and hold full copyright. They agree to publish this questionnaire as Supporting Information under the Creative Commons Attribution License (CC BY 4.0), which permits unrestricted use, distribution, and reproduction in any medium, provided the original authors and source are properly credited.

**Health Provider-Family Bidirectional Digital Communication Scale (HF-BDCS)**

Dear Medical Team and Family Member,

This scale aims to measure the experiences and perceptions of digital communication between medical staff and family members through digital communication methods. Your responses will help us improve future healthcare communication practices. Please answer each question based on your actual experience and honest feelings. All information collected will be used solely for academic research and will remain strictly confidential. Your responses will not be disclosed to any healthcare providers and will not affect the rights of your hospitalized family member in any way. Thank you for your time and participation.

Sincerely,
Department of Nursing, National Taiwan University Hospital
Yu-Chun Diao, RN

| Physician | Gender: □ Male □ Female  Age: ______ years  Education Level: □ Bachelor's □ Master's  Years of Experience (other hospitals + current): ______ years ______ months  Position Level: □ R1 □ R2 □ R3 □ CR □ VS |
| --- | --- |
| Nurse | Gender: □ Male □ Female  Age: ______ years  Education Level: □Bachelor's □ Master's  Years of Experience (other hospitals + current): _____ years ______ months  Position Level: □ N1 □ N2 □ N3 □ N4 |
| Family Member | Gender: □ Male □ Female  Age: ______ years  Education Level: □ Elementary □ Junior High □Senior High □ University □ Graduate School  Relationship to Patient: □ Spouse □ Child □ Grandchild □ Sibling □ Relative |

## The following items aim to understand your experience using digital communication methods to communicate with the medical team. Please indicate your level of agreement with each statement. Note: 'Digital communication methods' refer to any form of communication that is not face-to-face and is mediated by technology, such as LINE, phone calls, online video calls, or other communication software.

| Item | Strongly Disagree | Disagree | Neutral | Agree | Strongly Agree |
| --- | --- | --- | --- | --- | --- |
| 1.The hospital provides clear information on regulations regarding digital communication between providers and families. |  |  |  |  |  |
| 2. I know how to use the hospital's digital communication tools (e.g., LINE app, phone calls, online video calls, or other communication software). |  |  |  |  |  |
| 3.Digital communication methods eliminate the need for in-person hospital visits, making communication more convenient for both parties. |  |  |  |  |  |
| 4. Digital communication methods allow both parties sufficient time for medical communication outside of fixed visiting hours. |  |  |  |  |  |
| 5.Because digital communication methods do not require face-to-face interaction, they reduce stress for both parties. |  |  |  |  |  |
| 6. Digital communication methods facilitate communication between multiple family members and the health-care team. |  |  |  |  |  |
| 7. When changes in a patient’s condition require unexpected medical decisions, digital communication methods facilitate faster medical decision-making. |  |  |  |  |  |
| 8. Digital communication methods can be as effective as face-to-face communication. |  |  |  |  |  |
| 9. Through digital communication methods, both parties can adequately understand and provide information regarding the patient’s medical treatment during hospitalization. |  |  |  |  |  |
| 10. Digital communication methods enable both parties to express and understand the content of a message. |  |  |  |  |  |
| 11. Through digital communication methods, both parties can listen patiently to each other. |  |  |  |  |  |
| 12. Through digital communication methods, both parties can actively express care or feel cared for. |  |  |  |  |  |
| 13. Through digital communication methods, both parties can communicate with a positive attitude. |  |  |  |  |  |
